# Supplementary material for: Socioeconomic deprivation and suicide in Appalachia: The use of three socioeconomic deprivation indices to explain county-level suicide rates
Source: PLoS One. 2024 Nov 18;19(11):e0312373. doi: 10.1371/journal.pone.0312373 (PMC11573156; doi:10.1371/journal.pone.0312373)
Supplement: S1 Table — (DOCX) [file pone.0312373.s001.docx]

### **S1 Table. Socioeconomic Deprivation Index Domains & Items**

|  | **Domain** | **Item** |
| --- | --- | --- |
| **Townsend Deprivation Index** |  |  |
|  | Socioeconomic Status | - % of the civilian labor force 16 years of age or older who are unemployed |
|  | Housing Type &  Transportation | - % living in renter-occupied housing units - % of overcrowded housing units (number of occupants per room is greater than 1.01) - % of households without a car |
| **Social Deprivation Index** |  |  |
|  | Socioeconomic Status | - % of those 25 years or older with less than 12 years of education - % of those living below 100% the federal poverty level - % of the civilian labor force 16-64 years of age who are unemployed |
|  | Housing Type & Transportation | - % of those living in renter-occupied housing units - % of overcrowded housing units (number of occupants per room is greater than 1.01) - % of households without a car |
| **Social Vulnerability Index** |  |  |
|  | Socioeconomic Status | - Unemployment rate for those 16 years of age or older in civilian labor force - % of those living below 150% the federal poverty level - % of housing cost burdened households - % of those who are uninsured - % of those 25 years of age or older without a high school diploma |
|  | Housing Type & Transportation | - % of housing structures with 10 or more units - % of mobile homes - % overcrowded housing units (number of occupants per room is greater than 1.01) - % of households without a car - % of those living in group quarters |
|  | Household Characteristics | - % of those 65 years of age or older - % of those 17 years of age or younger - % of those living with a disability - % of single-parent households   % of those 5 years of age or older who speak English “less than well” |
|  | Racial & Ethnic Minority Status | - % of racial and/or ethnic minorities |
