# Supplementary material for: Socioeconomic deprivation and suicide in Appalachia: The use of three socioeconomic deprivation indices to explain county-level suicide rates
Source: PLoS One. 2024 Nov 18;19(11):e0312373. doi: 10.1371/journal.pone.0312373 (PMC11573156; doi:10.1371/journal.pone.0312373)
Supplement: S2 Table — (DOCX) [file pone.0312373.s002.docx]

### **S2 Table. Results from Backward Selection Strategy: Townsend Deprivation Index Items**

|  | Model 1  AIC=379.4 | | Model 2  AIC=377.88 | | Model 3  AIC=376.92 | |
| --- | --- | --- | --- | --- | --- | --- |
| Variable | β | p | β | p | β | p |
| Intercept | 3.88 | <0.01* | 3.90 | <0.01* | 3.90 | <0.01* |
| Rurality | -0.14 | 0.03* | -0.14 | 0.02* | -0.15 | 0.01* |
| Unemployed | 0.05 | 0.06* | 0.05 | 0.06* | 0.06 | 0.02* |
| Overcrowded Households | 0.03 | 0.02* | 0.03 | 0.02* | 0.03 | 0.02* |
| Household Without Vehicle | 0.02 | 0.54 | 0.03 | 0.31 | --- | --- |
| Living in Renter Occupied Housing | 0.01 | 0.68 | --- | --- | --- | --- |
| Models adjusted for rural classification based on the 2013 Rural-Urban Continuum Codes (RUCC); To account for small sample sizes, significance was set at alpha=0.1 | | | | | | |
