# Supplementary material for: Socioeconomic deprivation and suicide in Appalachia: The use of three socioeconomic deprivation indices to explain county-level suicide rates
Source: PLoS One. 2024 Nov 18;19(11):e0312373. doi: 10.1371/journal.pone.0312373 (PMC11573156; doi:10.1371/journal.pone.0312373)
Supplement: S3 Table — (DOCX) [file pone.0312373.s003.docx]

**S3 Table. Results from Backward Selection Strategy: Social Deprivation Index Items**

|  | Model 1  AIC=377.64 | | Model 2  AIC=375.68 | | Model 3  AIC=374.17 | |
| --- | --- | --- | --- | --- | --- | --- |
| Variable | β | p | β | p | β | p |
| Intercept | 3.38 | <0.01* | 3.38 | <0.01* | 3.45 | <0.01* |
| Rurality | -0.20 | <0.01* | -0.20 | 0.01* | -0.21 | <0.01* |
| Aged 25 or older w/Less Than 12 Years of Education | 0.01 | 0.17 | 0.01 | 0.13 | 0.02 | <0.01* |
| Overcrowded Households | 0.07 | 0.08* | 0.07 | 0.08* | 0.07 | 0.07* |
| Unemployed | 0.00 | 0.67 | 0.00 | 0.49 | --- | --- |
| Below 100% Poverty | 0.00 | 0.85 | --- | --- | --- | --- |
| adjusted for rural classification based on the 2013 Rural-Urban Continuum Codes (RUCC); To account for small sample sizes, significance was set at alpha=0.10 | | | | | | |
