# Supplementary material for: Socioeconomic deprivation and suicide in Appalachia: The use of three socioeconomic deprivation indices to explain county-level suicide rates
Source: PLoS One. 2024 Nov 18;19(11):e0312373. doi: 10.1371/journal.pone.0312373 (PMC11573156; doi:10.1371/journal.pone.0312373)
Supplement: Attachment — Submitted filename: Response letter 09.2.24.docx [file pone.0312373.s008.docx]

Angela Mendes Freitas

Academic Editor

*PLOS ONE*

Dear Dr. Mendes Freitas,

Thank you for the opportunity to submit a revised version of our manuscript titled, “Socioeconomic deprivation and suicide in Appalachia: The use of three socioeconomic deprivation indices to explain county-level suicide rates”, for consideration in PLOS ONE. We are grateful for the time and effort you dedicated to finding reviewers, and we truly appreciate your persistence in doing so. We would also like to express our sincere thanks to the reviewer for their thorough and insightful feedback, which has significantly strengthened the manuscript and ensured it meets the high standards of the journal.

We have provided a detailed point-by-point response to the reviewers’ comments and concerns on the following pages. All modifications to the manuscript are shown as tracked changes, and we have also included a clean version of the revised manuscript for your review.

We hope this revision meets the standards of *PLOS ONE* and is acceptable for publication. We look forward to your feedback and remain available to address any further questions or concerns you may have.

All my very best,

Erin Caswell

PhD Candidate

Department of Epidemiology and Biostatistics

School of Public Health

West Virginia University

G104C, HSC North

64 Medical Center Drive, Morgantown, WV 26501

Email: erin.caswell@hsc.wvu.edu

Phone: 540-532-8688

**Comments from Reviewer #1:**

Major revision

1) In the introduction, some main ideas appear repeatedly, mainly regarding the local evidence, while the main findings and gaps of similar studies developed for other regions of the world were not explored in depth. The topic has already been extensively studied for other regions, and the introduction should be aligned with current specific literature. In this sense, the local evidence could be reorganized into one or two paragraphs and more robust evidence on the relationship between the suicide rates and socioeconomic deprivation from studies developed for other regions should be considered in the introduction.

Response:

Thank you for your insightful feedback. We appreciate your suggestions on improving the depth and breadth of the introduction. In response, based on a robust review of literature, we have restructured the introduction to focus on the specific context of Appalachia, aligning with the aim of the study. This revision highlights the distinctive challenges faced by West Virginia in comparison to broader contexts, such as those observed in other areas of the United States. We, thus, aim to provide a clearer understanding of how socioeconomic deprivation impacts suicide rates specifically in West Virginia.

Pages 3-4 Lines 48-74.

*“……The Appalachian Region is comprised of thirteen states across the eastern United States and the region is known to have several social factors, such as lack of social capital, increased substance use, lower educational attainment levels, barriers to transportation, and substandard housing characteristics, such as overcrowding [2,3], that can be referred to in aggregate as socioeconomic deprivation. As the only state entirely within Appalachia, West Virginia reflects the profound socioeconomic deprivation that defines much of the region. [4] These factors collectively contribute to heightened vulnerability to suicide and other adverse outcomes—such as mental health disorders and substance abuse—by compounding stress stemmed from negative social and economic conditions. [5] This vulnerability is particularly pronounced in rural areas, where individuals face greater isolation and have fewer resources to manage mental health challenges. [6] Socioeconomic deprivation, exacerbated by West Virginia’s rural geography, has potentially contributed to a troubling rise in suicide rates.*

*Focusing research to West Virginia is critical not only because of its disproportionate impact on the state's residents but also because it underscores the broader challenges faced by the entire Appalachian Region. [2-4, 7] Consequently, Appalachian residents are 21% more likely to die by suicide than those in other parts of the United States [7], illustrating the vulnerabilities linked to the region’s widespread socioeconomic deprivation. West Virginia's unique geographical and socioeconomic context makes it essential to understand local conditions, as studies from other United States regions show that suicide risk factors can vary considerably based on location. For example, factors like urban density and economic disparities in metropolitan areas may be more influential in broader United States contexts. [8-9] Whereas, West Virginia’s high rates of rural isolation, limited access to healthcare, and persistent economic hardship create distinct challenges. [6] Although, socioeconomic deprivation is often measured through various indices that assess the association between deprivation and suicide, has yet to be evaluated specifically within the Appalachian West Virginia population. [10-13]*

*Given the region's unique social and economic landscape, there is a critical need to identify the specific factors that constitute socioeconomic deprivation in this area and determine their significant contributions to West Virginia’s elevated suicide rates…….”*

References:

Palomin A, Takishima-Lacasa J, Selby-Nelson E, Mercado A. Challenges and Ethical Implications in Rural Community Mental Health: The Role of Mental Health Providers. Community Ment Health J. 2023;59(8):1442-51.

Rogerson P, Yang J, Bagchi-Sen S. Recent geographic patterns in suicide in the United States. GeoJournal. 2024;89(1):19.

Mukherjee S, Wei Z. Suicide disparities across metropolitan areas in the US: A comparative assessment of socio-environmental factors using a data-driven predictive approach. PLoS One. 2021;16(11):e0258824.

Marshall J, Thomas, L., Lane, N.M., Holmes, G. M., Arcury, T. A., Randolph, R., Silberman, P., Holding, W., Villamil, l., Thomas, S., Lane, M., Latus, J., Rodgers, J., Ivey K. Health Disparities in Appalachia. 2017.

Mezzina R, Gopikumar V, Jenkins J, Saraceno B, Sashidharan SP. Social Vulnerability and Mental Health Inequalities in the "Syndemic": Call for Action. Front Psychiatry. 2022;13:894370.

2) In the following lines, the authors concluded that the differences found in the associations between poverty and suicide by previous studies were due to the poverty measure. However, many other factors could be influencing it (e.g., the geographic area, the method used to measure the association, etc.). Thus, it is necessary to explain how these studies are relatable and if there is evidence for the same area and using the same methods, for example.

Lines 74-77. “Therefore, studies examining the association between poverty and suicide using a single poverty measure have shown varied results depending on the metric used to capture poverty. For example, a few studies found significant inverse relationships between poverty and suicide [20-23], while others found no association [23-25]”.

Response:

Thank you for pointing out the need for a more nuanced discussion regarding the variability in findings from previous studies. To address this concern, we have revised the relevant section to include:

Pages 4-5 Lines 94-101.

*“Therefore, studies examining the association between poverty and suicide using a single poverty measure have shown varied results depending on the metric used to capture poverty. For example, a few studies found significant inverse relationships between poverty and suicide [23-26], while others found no association. [26-28] These discrepancies could stem not only from different poverty metrics used but also from other factors, including geographic variability, the methods employed to measure associations, and differing population characteristics. Hence the variability in findings reflects the multifaceted nature of socioeconomic deprivation and the context in which it is studied. [29]”*

References:

Lemmi V, Bantjes J, Coast E, Channer K, Leone T, McDaid D, et al. Suicide and poverty in low-income and middle-income countries: a systematic review. The Lancet Psychiatry. 2016;3(8):774-83.

3) In general, along the introduction, the authors often referred about the potential effects of “poverty” and “socioeconomic deprivation” on suicide. The attempt to distinguish these concepts was not clear (e.g., lines 70-74). It is necessary to define these two concepts, explain how they are related and rewrite the introduction accordingly, to give a better understanding of the problem. I think it is also important to define and explain the relationship between these two concepts and “vulnerability”, since one of the selected indices were the Social Vulnerability Index (SVI).

Response:

Thank you for highlighting the need for clarity in distinguishing between “poverty,” “socioeconomic deprivation,” and “vulnerability.” In response to your comment, we have revised the introduction to explicitly define and differentiate these concepts and explain their interrelationships.

Page 4. Lines 79-86.

*“Although often interconnected, it is crucial to differentiate between “poverty”, "socioeconomic deprivation," and "vulnerability" for identifying associations with suicide. Poverty refers specifically to a lack of financial resources, while socioeconomic deprivation encompasses a wider range of hardships, such as limited access to education, substandard housing, inadequate transportation, and diminished social capital. Unlike poverty, which focuses on income, socioeconomic deprivation provides a more holistic view by capturing multiple dimensions of material and social disadvantage. Vulnerability, on the other hand, reflects an increased susceptibility to adverse outcomes, such as suicide, due to the cumulative effects of socioeconomic deprivation. [17]”*

References:

Biswas S, Nautiyal S. A review of socio-economic vulnerability: The emergence of its theoretical concepts, models and methodologies. Natural Hazards Research. 2023;3(3):563-71.

4) In the section “Identifying Key Index Items”, lines 213-214, the authors mentioned that the final model, following the backward selection strategy, included the percentage of those without a high school diploma and other variables. According to the methods, items with the highest p-value should be excluded from the models one at a time until all items remained significant at the 0.10 level (lines 211-212). In this sense, the inclusion of the percentage of those without a high school diploma seems a mistake. The p-value of this variable (0.11) is quite close to the significant level of reference, but its inclusion needs to be justified.

Response: Thank you for your feedback. We acknowledge that this approach may require further clarification. When conducting the backward selection, we considered not only the significance of each item but also the AIC. For instance, after removing education from model 5, the AIC increased, indicating a worse model fit. Therefore, education was retained in the final model to ensure the best fit. We have now included the following rationale for including the percentage of individuals without a high school diploma in the model within the results and methods sections.

Page 8 Lines 174-177

*“Finally, a backward selection modeling strategy accounting for overall model improvement in AIC identified key items for examining suicide rates in each index. The significance level was set to 0.10 to account for small sample sizes and identify trends among key items. If removing an item increased model AIC significantly, the prior model was selected as the preferred model.”*

Page 13. Lines 249-252

*“Although the percentage of individuals without a high school diploma had a p-value of 0.11, slightly above the threshold of .10, it was retained because its removal did not improve the Akaike Information Criterion (AIC). This inclusion ensured the best model fit. [50]”*

Reference:

Chowdhury MZI, Turin TC. Variable selection strategies and its importance in clinical prediction modelling. Fam Med Community Health. 2020;8(1):e000262.

Minor revision

1) It is necessary to mention the area that is being compared to the Appalachian Region in the following lines:
Lines 48-49. “The Appalachian Region has an urgency to reduce suicide rates, as its residents are 21% more likely to die by suicide”.

Response: Thank you for your comment. I agree the original wording was unclear. I have revised the sentence for clarity:

Page 3 Lines 62-63

*“Appalachian residents are 21% more likely to die by suicide than those in other parts of the United States [7].”*

2) There is duplicate information in the following lines:
Lines 101-103. “Additional cases were those identified using the classification codes that represent accidental poisonings 101 (X40-X43) and poisoning of undetermined intent (Y10-Y15). X40-X43 (accidental poisoning and Y10- 102 Y15 (poisoning of undetermined intent)”.

Response: Thank you for highlighting this duplication. The repeated line, “X40-X43 (accidental poisoning) and Y10-Y15 (poisoning of undetermined intent),” has been removed.

3) In the “Suicide Estimates”, it is important to mention the source of the population estimates used to calculate the suicide rates. Only in Table 1 it was possible to conclude that it was obtained from the same database as suicide cases.

Response: Thank you for your helpful suggestion. I have revised the final sentence in the “Suicide Estimates” section to clarify the source of the population estimates:

Page 5. Line 129-130.

*“Population estimates were also obtained from the WONDER database, and the analysis was restricted to adults aged 18 and older.”*

4) In the “Three Socioeconomic Deprivation Indices”, it is important to cite the first publications of the methodologies used to calculate the selected indices. As the different deprivation/vulnerability indices are central elements of the study and different methods can produce different results of deprivation, a brief description of how the scores of these indices are obtained is also needed in this section.

Thank you for your valuable feedback. In response to your suggestion, we have cited the original publications for the methodologies used to calculate each of the three socioeconomic deprivation indices: the Townsend Deprivation Index, Social Deprivation Index, and Social Vulnerability Index. Additionally, we have included a brief description of how scores for each index were derived, highlighting the methodologies and calculation processes. These updates aim to clarify the construction of these indices and acknowledge that different methods can yield varying results. The following lines have been revised and added to the manuscript:

Page 6 Line 138-139

*“County-level Townsend Deprivation Index scores were manually created following the methodology outlined in the original publication. [38]”*

Page 7 Lines 144-152

*“For the Social Deprivation Index, a composite measure was created using demographic variables from the ACS. These variables were standardized and weighted based on factor analysis to include only those strongly associated with deprivation. [30, 31] To construct the Social Vulnerability Index, ACS variables were ranked for all United States census tracts, with each variable assigned a percentile rank. For most variables, higher ranks indicated greater vulnerability, except for per capita income, where higher values indicated less vulnerability. Percentile ranks were calculated for each variable and domain. Finally, an overall percentile rank was granted for each census tract. [40] County-level scores for the Social Deprivation Index and the Social Vulnerability Index were readily available from their host organization. [30, 32]”*

References:

**(TDI Original Methodology) –**

Townsend P. Deprivation. Journal of Social Policy. 1987;16:125-46. Available from: https://doi.org/ 10.1017/S0047279400020341

**(SDI Original Methodology) –**

Butler DC, Petterson S, Phillips RL, Bazemore AW. Measures of Social Deprivation That Predict Health Care Access and Need within a Rational Area of Primary Care Service Delivery. Heath Services Research 2013;48(2):5389-559. Available from: https://www.ncbi.nlm.nih.gov/pubmed/22816561

**(SVI Original Methodology) –**

Flanagan B, Gregory E, Hallisey E, Heitgerd J, Lewis B. A Social Vulnerability Index for Disaster Management. Journal of Homeland Security and Emergency Management. 2011;8.

5) In the “Statistical analysis”, lines 125-126, it is important to cite the reference used for the data suppression.

Response: Thank you for your suggestion. I have now included the reference for data suppression in the manuscript. According to the CDC Wonder's Assurance of Confidentiality constraints, counts less than 10 must be excluded.

Lines 157-159

Reference:

Centers for Disease Control and Prevention. Multiple Cause of Death 1999–2020.

6) In the “Statistical analysis”, lines 130-131, it is important to cite the references that justified the choice for the model (negative binomial model). I am not sure if the negative binomial regression can handle underdispersion, specifically. Furthermore, did the data present overdispersion or underdispersion? That wasn't clear.

Response: I appreciate your comment. We selected the negative binomial regression for this analysis as it is better suited to handle overdispersion compared to Poisson regression. We used the ‘check_overdispersion’ function in R, which indicated that while the dispersion ratios ranged from 1.08 to 1.14—suggesting a slight potential for overdispersion—the test did not find significant overdispersion. Despite this, we chose the negative binomial model to ensure a more conservative approach and account for any minor deviations from the Poisson assumptions. I have now included the following in the limitations section and provided the reference in both the limitations and statistical analysis sections:

Page 7 Line 163.

“Negative binomial regressions were chosen to account for the expected non-normal distribution of suicide rates and for overdispersion. [46]”

Page 17 Lines 340-342.

“*Although tests indicated that there was no significant overdispersion in our data, we selected the negative binomial regression model to provide a more conservative approach and ensure robustness in our analysis. [46]”*

Reference:

Overdispersion. In: Hilbe JM, editor. Negative Binomial Regression. 2 ed. Cambridge: Cambridge University Press; 2011. p. 141-84.

7) In the “Statistical analysis”, lines 131-133, it is important to cite the references of suicide research used to justify why the models were adjusted for rurality.

Response: Thank you for your suggestion. I have now included a reference to support the adjustment for rurality in the models.

Line 165.

Reference:

Pritchard TR, Buckle JL, Thomassin K, Lewis SP. Rural Suicide: A Systematic Review and Recommendations. Clinical Psychological Science. 2024;0(0).

8) In the “Descriptive Statistics”, the authors mention that the socioeconomic deprivation scores are not comparable between indices (lines 149-150). The interpretation of the results could be facilitated by transposing the scores of the 3 indices into the same scale of values.

Response: Thank you for this insightful comment. We appreciate the suggestion to transpose the scores of the three indices to the same scale of values to facilitate interpretation. However, after careful consideration, indices were not standardized for the following reasons:

1. Preservation of Original Meaning: Each index was developed with specific constructs and measurement scales in mind, reflecting different dimensions of socioeconomic deprivation and vulnerability. Standardizing the scores would remove the original context and meaning of these indices, potentially making the results less interpretable in practical terms. For example, a high score on one index may have a different practical implication than a high score on another, even if they are standardized. Additionally, two of the three indices produce percentiles scores which does not support a standardization approach.
2. Risk of Misleading Comparisons: Standardizing the indices could lead to comparisons that may not accurately reflect the true relationships between the indices and the outcome of interest (suicide rates). The different scales and underlying constructs of these indices are inherently distinct and transposing them to a common scale might obscure these differences, leading to potentially misleading interpretations.
3. Focus on Absolute Values: Our analysis prioritizes understanding the impact of absolute levels of deprivation as measured by each index. By maintaining the original scales, we aim to provide clear, context-specific insights that can be directly applied to policy and intervention strategies.
4. Alignment with Literature: Our approach is consistent with existing literature, where these indices are often analyzed in their original scales to retain their unique contributions to the understanding of socioeconomic deprivation and its effects. This allows our findings to be more easily compared with other studies.

We have added the following clarification in the revised manuscript:

Page 8. Lines 182-186

*“While socioeconomic deprivation scores are not comparable between indices due to their distinct constructs and scales – standardizing these scores could obscure their original context and meaning due to inclusion of percentiles– the level of deprivation between counties can still be interpreted from their respective county index scores [49]. “*

Reference:

Phillips RL, Liaw W, Crampton P, Exeter DJ, Bazemore A, Vickery KD, et al. How Other Countries Use Deprivation Indices—And Why The United States Desperately Needs One. Health Affairs. 2016;35(11):1991-8.

9) In the lines 160-162, the following information does not coincide with the data shown in Table 2: “The lowest model AIC was found in the Social Vulnerability Index (370.20), followed by the Townsend Deprivation Index (374.52) and Social Deprivation Index (374.52)”.

Response: Thank you for bringing this to my attention. After rerunning the analysis and

reviewing the table, I found that the Social Deprivation Index's AIC should have been 381.29. I have updated the sentence to:

Page 10 Lines 194-196

“The lowest model AIC was found in the Social Vulnerability Index (370.20), followed by the Townsend Deprivation Index (374.52) and Social Deprivation Index (381.29).”

10) The scale of the map must be inserted in Figure 1.

Response: Thank you. We have revised Figure 1 to incorporate a scale bar to the bottom-right corner of the map.

11) In lines 165-166, it seems better to exclude the following information: “When compared to the reference map”. The conclusion does not seem to result from a comparison with the reference map.
“When compared to the reference map, the map of suicide rates predicted by the Social Deprivation Index demonstrated the least variability in suicide rates among counties (30-43 deaths per 100,000 people)”.

Response: Thank you. I have removed the phrase and revised the sentence to:

*Page 10. Lines 199-200*

*“The map of suicide rates predicted by the Social Deprivation Index demonstrated the least variability in suicide rates among counties (30-43 deaths per 100,000 people).”*

12) In the section “Examining Individual Index Items”, the authors forgot to mention the significant value observed for the “Aged 25 or older with Less Than 12 Years of Education (%)” variable, which composes the SDI. A variable related to education was presented in 2 out of the 3 indices (the SDI and SVI). In both cases, the variable related to education presented a significant value (Table 3), but it was ignored in the text, in which the authors only mentioned the other significant variables.

Response: Thank you for highlighting this oversight. In the revised section “Examining Individual Index Items,” I have included the significant findings for the variable “Aged 25 or older with Less Than 12 Years of Education” in the SDI.

Page 11 Lines 214-218.

*“Education was a significant item shared between the Social Deprivation Index and the Social Vulnerability Index. In the Social Deprivation Index, the percentage of those aged 25 years or older with less than 12 years of education exhibited a positive linear relationship with suicide rates (β=0.02, p<0.01). Similarly, the Social Vulnerability Index showed a significant trend for the percentage of those without a high school diploma (β=0.02, p<0.01).”*

13) In the section “Identifying Key Index Items” (lines 208-211), the authors forgot to mention the percentage of those without a high school diploma (β=0.02, p<0.01).

Response: Thank you, the education variable should have been included in this sentence.

Page 12 Lines 243-246

*“In the Social Vulnerability Index (Table 4), the first model included all significant items: unemployment rate, percent living below 150% of the federal poverty level, percent of overcrowded housing units, percent of housing cost-burdened households, percent of ethnic and racial minorities, percent of those living with a disability and percent of those without a high school diploma”*

14) In the discussion (lines 300-301), the authors mentioned that multicollinearity could be present in the analyses but did not appear to be substantial based on the variance inflation factors. It is recommended to show the maximum value observed for multicollinearity.

Response: Thank you for your valuable feedback. To address your comment, we have updated the discussion to include the maximum variance inflation factor (VIF) observed in our analysis. The sentence in the limitations has been revised to:

Page 17 Lines 338-339

*“Multicollinearity could be present in our analyses but did not appear to be substantial based on the variance inflation factors (VIF), with the highest VIF observed being 1.13 in our analyses.”*

15) In Supporting Information (S1_Table), it is recommended to show which age group was considered to calculate the unemployment rate of the SVI.

Response: Thank you for pointing this out. We have revised Supporting Information (S1_Table) to specify the age group considered for calculating the unemployment rate in the Social Vulnerability Index (SVI).
