# Supplementary material for: Socioeconomic deprivation and suicide in Appalachia: The use of three socioeconomic deprivation indices to explain county-level suicide rates
Source: PLoS One. 2024 Nov 18;19(11):e0312373. doi: 10.1371/journal.pone.0312373 (PMC11573156; doi:10.1371/journal.pone.0312373)
Supplement: Attachment — Submitted filename: Response letter_09202024.docx [file pone.0312373.s009.docx]

Angela Mendes Freitas

Academic Editor

*PLOS ONE*

Dear Dr. Mendes Freitas,

Thank you for the opportunity to resubmit our revised manuscript, “Socioeconomic Deprivation and Suicide in Appalachia: The Use of Three Socioeconomic Deprivation Indices to Explain County-Level Suicide Rates,” for consideration in PLOS ONE. We appreciate the reviewer's detailed and insightful feedback, which has been crucial in enhancing the manuscript.

In this revision, we have addressed the reviewer's concerns by including the dataset and codebook to ensure transparency and reproducibility of our results. Additionally, we have clarified the distinction between "poverty" and "socioeconomic deprivation." The manuscript now explicitly defines "poverty" as a lack of financial resources, typically measured by income, while "socioeconomic deprivation" is described as a broader concept that includes poverty but also encompasses additional material and social disadvantages.

We have included a detailed point-by-point response to the reviewer’s comments on the following pages. The modifications to the manuscript are shown in tracked changes, and we have also provided a clean version for your review.

We hope these revisions align with the journal’s standards and look forward to your feedback. Please let us know if there are any further questions or issues.

All my very best,

Erin Caswell

PhD Candidate

Department of Epidemiology and Biostatistics

School of Public Health

West Virginia University

G104C, HSC North

64 Medical Center Drive, Morgantown, WV 26501

Email: erin.caswell@hsc.wvu.edu

Phone: 540-532-8688

Reviewer #1: The authors have attempted to address all comments from the previous round of review. However, I believe that the following two points still require major revisions:

1) In the "Supporting information - Manuscript Data", the following file does not present the data: “tdisuicide_final_ruralclass.xlsx”. In addition, all files are missing the data dictionary.

Thank you for bringing this to our attention. We have thoroughly reviewed the files to ensure all data are complete and have included the variable codebook on the first sheet of each Excel document.

2) The attempt to differentiate "poverty" and "socioeconomic deprivation" was not clear. The authors presented their own definitions, but it is important to present the references that guided the choice of these definitions. Furthermore, there is still inconsistent information in the text, for example between the following excerpts:

Page 4. Lines 80-84. "Poverty refers specifically to a lack of financial resources, while socioeconomic deprivation encompasses a wider range of hardships, such as limited access to education, substandard housing, inadequate transportation, and diminished social capital. Unlike poverty, which focuses on income, socioeconomic deprivation provides a more holistic view by capturing multiple dimensions of material and social disadvantage".

Page 4. Lines 88-94. "For example, socioeconomic deprivation has been assessed using readily available poverty measures such as unemployment, education level, and income. [13] Studies in the United States have predominantly relied on income to measure poverty. [18-21] However, poverty assessed solely by income only indirectly measures socioeconomic deprivation. Poverty is a complex construct comprised of many factors that income alone does not fully capture, such as lack of material resources and overall standard of living. [20] These standalone measures of poverty may not capture socioeconomic deprivation comprehensively and accurately. [22]".

We agree that there is a need for clarity regarding the distinction between poverty and socioeconomic deprivation. In our study, "poverty" specifically refers to a lack of financial resources, typically measured by income levels. This definition aligns with the conventional use of poverty metrics [17]. On the other hand, "socioeconomic deprivation" is a broader construct that includes poverty but extends beyond it to encompass various dimensions of material and social disadvantage. These additional factors include limited access to education, substandard housing, inadequate transportation, and/or diminished social capital [18]. This broader perspective allows us to capture a more comprehensive view of poverty that goes beyond mere financial constraints.

**The revised text now reads as:**

*Page 4. Lines 82-88. “Poverty has been generally used to refer to a lack of financial resources, typically measured by income. [17] Socioeconomic deprivation, however, is a broader concept that includes poverty but also captures additional material and social disadvantages such as limited access to education, substandard housing, inadequate transportation, and/or diminished social capital. [18] Vulnerability, on the other hand, reflects an increased susceptibility to adverse outcomes, such as suicide, due to the cumulative effects of socioeconomic deprivation [17, 19].”*

*Pages 4-5. Lines 89-95. “Different measures have been used to assess socioeconomic deprivation while examining its association with suicide. For example, socioeconomic deprivation has been assessed using measures such as unemployment, education level, and income. [13] Studies in the United States have predominantly relied on income to measure poverty. [20-23] Consequently,, income as a standalone measure of poverty may not capture socioeconomic deprivation comprehensively and accurately. [24] By focusing solely on income, important contributors to social and material disadvantage are often overlooked, leading to an incomplete assessment of overall socioeconomic deprivation.”*

Newly Added References:

17 Ouoya Z. Poverty And Vulnerability To Poverty: Conceptual Overview, Measurements And Causes. International Journal of Scientific and Research Publications (IJSRP). 2021;11:378-94.
18 Gweshengwe B, Hassan NH. Defining the characteristics of poverty and their implications for poverty analysis. Cogent Social Sciences. 2020;6(1):1768669.
